# Neuroinflammation in Multiple System Atrophy: Response to and Cause of α-Synuclein Aggregation

**DOI:** 10.3389/fncel.2015.00437

**Published:** 2015-11-12

**Authors:** Bruno Di Marco Vieira, Rowan A. Radford, Roger S. Chung, Gilles J. Guillemin, Dean L. Pountney

**Affiliations:** ^1^Menzies Health Institute Queensland, Griffith UniversityGold Coast, QLD, Australia; ^2^Department of Biomedical Sciences, Faculty of Medicine and Health Sciences, Macquarie UniversitySydney, NSW, Australia

**Keywords:** multiple system atrophy, α-synuclein, neuroinflammation, astrocytes, microglia

## Abstract

Multiple system atrophy (MSA) is a progressive neurodegenerative disease presenting with combinations of autonomic dysfunction, parkinsonism, cerebellar ataxia and/or pyramidal signs. Oligodendroglial cytoplasmic inclusions (GCIs) rich in α-synuclein (α-syn) constitute the disease hallmark, accompanied by neuronal loss and activation of glial cells which indicate neuroinflammation. Recent studies demonstrate that α-syn may be released from degenerating neurons to mediate formation of abnormal inclusion bodies and to induce neuroinflammation which, interestingly, might also favor the formation of intracellular α-syn aggregates as a consequence of cytokine release and the shift to a pro-inflammatory environment. Here, we critically review the relationships between α-syn and astrocytic and microglial activation in MSA to explore the potential of therapeutics which target neuroinflammation.

## Introduction: Multiple System Atrophy and α-Synuclein

Multiple System Atrophy (MSA) is a complex progressive neurodegenerative disease which affects 3.4–4.9 cases/100,000 with 0.6–0.7/100,000 new cases each year. It has no significant gender bias with disease onset typically over the age of 60 and a mean survival of ~7.9 years following diagnosis (Watanabe et al., 2002; Wenning et al., 2013; Longo et al., 2015). Although there are no strong genetic determinants, studies have associated MSA cases with the H1 haplotype of *MAPT*, also linked to tauopathies such as Progressive Supranuclear Palsy (PSP; Vilariño-Güell et al., 2011), *COQ2* loss of function linked mutations (Multiple-System Atrophy Research Collaboration, 2013) and hexanucleotide repeat expansions in *C9orf72* (also associated with Amyotrophic Lateral Sclerosis and Frontotemporal Dementia; Goldman et al., 2014). Clinically, a predominance of Parkinsonism (MSA-P) or Cerebellar Ataxia (MSA-C) plus a heterogenous combination of pyramidal signs, autonomic and urogenital dysfunctions may be detected (Longo et al., 2015). Due to this complex phenotype, definite MSA diagnosis requires autopsy to detect glial cytoplasmic inclusions (GCIs) immunopositive for α-synuclein (α-syn) and neurodegeneration in striatonigral or olivopontocerebellar structures (Lantos, 1998; Trojanowski and Revesz, 2007; Gilman et al., 2008).

Alpha-synuclein is a 14.4 kDa protein of predominant neuronal pre-synaptic location where it is believed to chaperone the assembly of synaptic vesicles for exocytosis via interaction with synaptotagmin (SNARE complex component) and has characteristic conformational plasticity. It normally exists as a soluble monomer/tetramer in equilibrium with a membrane-bound α-helical multimer (Narayanan and Scarlata, 2001; Tong et al., 2009; Burré et al., 2014). However, for reasons not yet fully elucidated, α-syn may misfold into abnormal dimers, oligomers, or fibrils/protofibrils that aggregate and constitute the pathological hallmark of several neurodegenerative conditions, including Parkinson’s disease (PD) and Dementia with Lewy Bodies (DLB), where they primarily occur in neurons (McKeith et al., 2005; Shulman et al., 2011). As mature human oligodendrocytes do not express α-syn normally (Miller et al., 2005), the origin of α-syn glial aggregates in MSA is unclear, whether as a consequence of primary oligodendrogliopathy followed by neuronal degeneration or a neuronal α-synucleinopathy leading to glial inclusions (Nishie et al., 2004; Wenning et al., 2008). Thus, it has been hypothesized that intercellular transmission of α-syn might be occurring via mechanisms such as endocytosis, direct penetration, micropinocytosis, pore formation, nanotube tunneling or diffusion (Ubhi et al., 2011; Konno et al., 2012). Nevertheless, the proposed mechanisms of release and subsequent cellular uptake suggest that at α-syn pathology transmission may occur in a putative prion-like manner (Prusiner et al., 2015).

**Figure 1:**
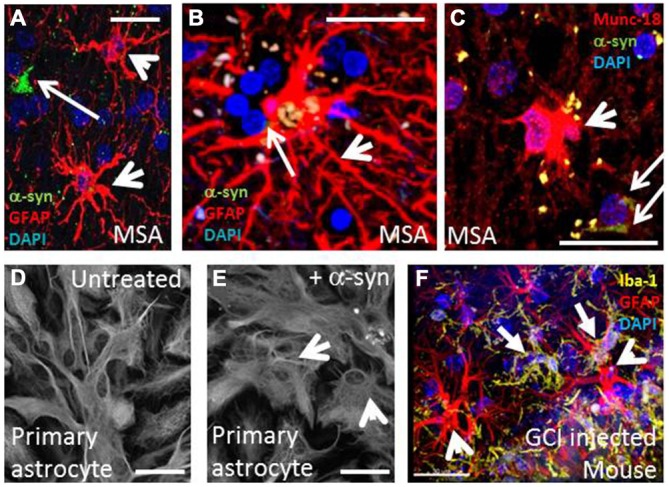
**Multiple system atrophy is characterized by widespread oligodendroglial α-syn inclusion bodies, astrogliosis and microgliosis. (A,B)** MSA putamen **(A)** and visual **(B)** showing activated astrocytes (arrowheads, GFAP, red) in close proximity to GCIs (arrows, α-syn, green). **(C)** A subset of activated astrocytes are also intensely immunopositive for the exocytic vesicle marker, munc18 (Radford et al., 2015). **(D,E)** Rat primary astrocyte cells adopt activated morphology when treated with α-syn. **(E)** compared to control cells **(D)** Scale bars, 20 μm. **(F)** Frequent activated microglia (solid arrow, yellow, Iba-1) and activated astrocytes (red, GFAP) occur near to the site of GCI injection in unilateral-lesioned mice (Radford et al., 2015). Scale bar, 30 μm.

In addition to α-syn rich GCIs as the central pathological feature, MSA also exhibits neuronal loss and strong neuroinflammation which both correlate with the density of inclusions and disease duration (Gai et al., 2003; Ozawa et al., 2004; Ahmed et al., 2012) as well as expression of inflammatory markers (Chen et al., 2015). Neuroinflammation is a dynamic response that involves changes in glial cell morphology, number, function and concomitant production of signaling molecules (O’Callaghan et al., 2008; Shastri et al., 2013). In the context of neurodegenerative diseases, persistent intra- and extracellular imbalances (such as those caused by misfolded proteins, oxidative stress, and neuronal death) are known to trigger and chronically perpetuate this response, which is dominated by microglia and astrocytes (Takeuchi, 2013). Gliosis is the term that indicates the phenotypic changes of glia and is exemplified in Figures 1A–C, where activated astrocytes and microglia co-localize with GCI pathology. This manuscript explores the role of α-syn and its relationship to neuroinflammation mediated by astrocytes and microglia in MSA.

## α-Synuclein Toxicity and Spreading

That α-syn is the pathogenic root of neurodegeneration in MSA has been given further credence by the discovery of *SNCA* mutations in α-synucleinopathies (mostly PD), but where G51D and A53E α-syn mutations have been described in patients with atypical parkinsonism and may overlap with MSA (Polymeropoulos et al., 1997; Krüger et al., 1998; Zarranz et al., 2004; Lesage et al., 2013; Proukakis et al., 2013; Pasanen et al., 2014). In MSA, it remains unclear which α-syn form/s is the principal mediator of toxicity but their compact and insoluble structure allows them to resist intracellular cleavage, accumulate and disrupt otherwise normal downstream processes (e.g., the ubiquitin-proteasome system, synaptic exocytosis, mitochondrial metabolism and ER-Golgi transport; Burré et al., 2015). Multiple modes of α-syn toxicity are reviewed elsewhere, including membrane permeabilization by annular oligomers and disrupting protein degradation pathways by inhibition of the proteasome and autophagy (Cuervo et al., 2004; Winner et al., 2011; Daturpalli et al., 2013; Roberts and Brown, 2015), but it is accepted that this α-syn-induced dysfunction can lead to the death of central nervous system (CNS) cells that defines neurodegeneration (Radford et al., 2014).

In addition, the exchange of amorphous α-syn between neurons and glial cells via exo- and endocytosis characterizes the cell-to-cell transmission and uptake that may ultimately lead to pathology spread (Reyes et al., 2014), with evidence for unconventional exocytosis (independent from ER-Golgi) and exosomes as mechanisms of release (Emmanouilidou et al., 2010; Jang et al., 2010). Key studies have recently provided evidence for α-syn transmission to occur in a prion-like manner. Thus, α-syn isolated post-mortem from MSA cases was transferred to HEK cells (modified to express the A53T mutation and tagged with yellow fluorescent protein, namely α-syn 140*A53T-YFP), that were then found to increase aggregate formation and increase YFP aggregate expression from the infected single-cells group, when compared to untransfected group (indicating a *de novo* prion formation in the transfected group; Woerman et al., 2015). In line with these findings, when brain extracts from MSA cases were injected intracerebrally in transgenic mice (M83 carrying the A53T mutation, namely tgM83), phenotypic changes (e.g., dysmetria and circling behavior) manifested 100–150 days post-inoculation in the homozygous group and pathological aggregates of phosphorylated α-syn and astrogliosis were detected in regions including brainstem. Interestingly, tgM83 mice inoculated with PD homogenates did not exhibit specific α-syn deposition or manifest clinical alterations significantly different from those of controls (Prusiner et al., 2015). These findings provide experimental evidence for MSA as a possible prion disease, with different α-syn strains being able to spread and promote tissue pathology in contrast with those of PD.

## Neuroinflammation: Astrocytes, Microglia and α-Synuclein in MSA

Astrocytes are key players in CNS homeostasis and pathology and are involved in a wide array of functions including modulating CNS immunity and inflammation, synaptic pruning and degradation of neuronal organelles (De Keyser et al., 2008; Sofroniew and Vinters, 2010; Chung et al., 2013; Hostenbach et al., 2013; Davis et al., 2014; Ben Haim et al., 2015). In MSA, the influence of α-syn on astrogliosis has been investigated by several studies. Treatment of primary astrocytes with α-syn promoted astrogliotic changes, as shown in Figures 1D,E (Radford et al., 2015). In astrocytes transfected with an inhibitor of endocytic vesicle formation (dominant negative dynamin-1 K44A mutant) and co-cultured with neuron-derived cell lines expressing α-syn, endocytosis was shown as a mechanism for direct uptake, which strongly correlates with the production of cytokines (such as IL-1α, -1β, -6, -18), colony-stimulating factors (CSF-1, -2, -3), and chemokines [(CCL-3, -4, -5, -12, -20), (CXCL-1, -2, -5, -10, -11, -12, -16)] (Lee et al., 2010). Moreover, in transgenic mice overexpressing oligodendroglial α-syn, exposure to oxidative stress (using the mitochondrial inhibitor 3-nitropropionic acid) led to astrogliosis and degeneration in close proximity to GCIs (Stefanova et al., 2005). Accordingly, the morphometric analysis of human cases and mouse models of MSA reveals that the degree of astrogliosis increased with proximity to α-syn deposits, as seen in Figures 1A,B (Song et al., 2009; Radford et al., 2015).

Microglia account for approximately 10% of all brain cells and derive from a primitive myeloid lineage of macrophages that migrate to cerebral regions during intrauterine life, after which they are distributed unevenly throughout the brain hemispheres, mostly concentrating in the hippocampus, basal ganglia and substantia nigra (Prinz et al., 2011). Normally, in the healthy brain, resident microglia adopt a resting (surveillant) phenotype, which is maintained by feedback of signaling molecules such as neuronal fractalkine and astrocytic glial-derived neurotropic factor, with perturbations of homeostasis triggering microglial activation into effector phenotypes, namely M1 and M2 (Tang and Le, 2015). This requires interaction of the noxious stimulus with immune response receptors such as complement factors, pattern recognition receptors and scavenger receptors (Husemann et al., 2002; Scheffel et al., 2012). Once activated, the M1 phenotype produces pro-inflammatory and cytotoxic molecules, such as TNF-α, IL-6, IL-1β, superoxide, NO, reactive oxygen species (ROS) and excitatory amino acids, which can induce more neuronal damage and progression of cellular dysfunction (Kettenmann et al., 2011). The other activated state, M2, also manifests phagocytic activity, but performs anti-inflammatory responses through release of IL-10 and transforming growth factor beta (TGF-β). It may also be induced by anti-inflammatory cytokines (e.g., IL-13 and IL-14) and acts in tissue repair via release of growth factors such as major histocompatibility complex 5, monocyte chemoattractant protein 1 and insulin-like growth factor 1 (Colton and Wilcock, 2010; Welser-Alves and Milner, 2013).

Morphologically, cellular hypertrophy and branching are the most commonly described changes in microgliosis which, depending on nature and intensity of the damage, may be detected as early as minutes to hours after acute injuries, with rapid process extension occurring in an ATP-dependent manner through P2Y12 receptors (Jensen et al., 1999; Davalos et al., 2005; Nimmerjahn et al., 2005; Parkhurst and Gan, 2010). In primary mesencephalic neuron-glia culture systems, extracellular α-syn was shown to be directly phagocytosed by microglia producing microgliosis, upregulation of NADPH oxidase and secretion of ROS, enhancing neurodegeneration (Zhang et al., 2005). Microgliosis can be identified clustering around α-syn years to decades after α-syn accumulation (Ishizawa et al., 2004; Graeber and Streit, 2010), or colocalizing with α-syn-rich neurons after direct stereotactic injection of α-syn ribbons or fibrils (Peelaerts et al., 2015).

In the extracellular environment, the abnormal presence of α-syn can be sensed and internalized by glial cells, leading to a cascade of reactive gliosis, secretion of pro-inflammatory cytokines and subsequent cell recruitment; characterizing the amplification of a localized deposit of protein. Localized micro- and astrogliosis resulted from injection of purified GCI material into mouse medial forebrain bundle after 23 days, as shown in Figure 1F (Radford et al., 2015). In microglia, pathogen pattern recognition receptors in the membrane surface enable the initial identification of foreign structural motifs on multiple arrays of pathogens (in the case of infectious disease), but they are also capable of recognizing changes in homeostatic cellular conditions and endogenous molecules, such as misfolded proteins in neurodegenerative diseases (Stefanova et al., 2005; Block et al., 2007). In particular, the Toll-like receptors (TLRs) 2 and 4 are known to interact with α-syn. In a cellular model, purified microglial cultures from brains of wild type (TLR4^+/+^) and deficient (TLR4^−/−^) postnatal mice were treated with wild-type and abnormal α-syn forms (fibrillar, truncated). The results revealed prolific microgliosis in the TLR4^+/+^ groups, increased phagocytic activity, upregulation of nuclear factor-kappa B (NF-κB), and increased production of CXCL1, IL-6, and TNF-α. Furthermore, TLR4-deficient microglia showed a reduced production of ROS upon α-syn treatments. In line with these findings, both human cases and transgenic mouse models of MSA also exhibit upregulation of TLRs (Béraud et al., 2011; Brudek et al., 2013; Fellner et al., 2013).

**Figure 2:**
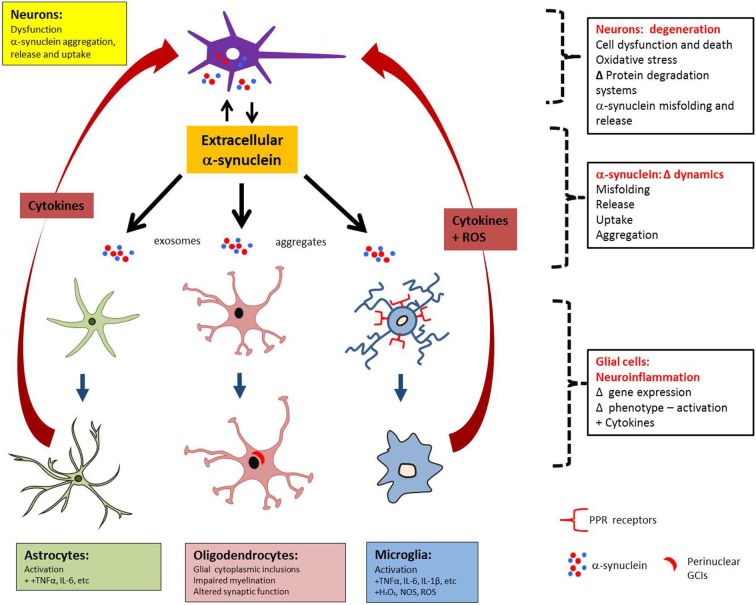
**MSA pathology may spread between anatomically connected regions as a result of reciprocal rounds of α-syn release and neuroinflammation.** Neuronal dysfunction can lead to α-syn aggregation and release of α-syn aggregates, which can then interact directly with astrocytes and microglia to mediate activation. In turn, the release of pro-inflammatory factors by activated glia can act back on neurons to cause stress, thereby stimulating the formation and release of additional α-syn aggregates.

The temporal relationship between astro- and microgliosis in MSA is still poorly understood, as preclinical studies cannot reproduce the long timescale of MSA pathogenesis. Clinical radiology could overcome this limitation but, to date, tagging neuroinflammation with specific markers (Gerhard et al., 2003) has not been performed (Schrag et al., 1998; Schocke et al., 2002; Ozawa et al., 2004; Watanabe et al., 2004; Brooks et al., 2009; Chandran and Stoessl, 2014). Despite limitations related to preclinical and clinical assessment of neuroinflammation, it may be that, because glial cells express no or very little α-syn, glial uptake of α-syn occurs and α-syn triggers the neuroinflammatory process, which may then operate in waves of incremental feed-forward damage. This process, combined with the prion-like behavior of α-syn, implicates the role of neuroinflammation in worsening/perpetuating MSA. Figure 2 represents schematically the interplay between α-syn aggregation and release, astrogliosis and microgliosis and the feedback of pro-inflammatory factors that may in turn result in additional neuronal stress.

It is known that extracellular α-syn may directly act upon astrocytes, microglia and oligodendrocytes. However, to date, there is a lack of studies specifically addressing gliosis as a trigger for α-syn misfolding and/or release (Croisier et al., 2005). Because of their role in surveillance and in reaction to pathogens, microglia may exert an indirect effect on α-syn by secreting a variety of toxic factors, which disrupt basic intracellular protein degradation systems and ultimately affect α-syn dynamics (Fellner and Stefanova, 2013). For example, it is known that α-syn oxidation and nitration inhibits chaperone-mediated autophagy (CMA; Kiffin et al., 2004; Martinez-Vicente et al., 2008; Xilouri et al., 2009, 2013) and that α-syn phosphorylation alters macroautophagy (Tenreiro et al., 2014) which, unlike CMA, can eliminate larger protein species, such as oligomers and aggregates (Engelender, 2012; Tanik et al., 2013). Moreover, neuron-glia cultures treated with lipopolysaccharide (LPS), a potent stimulator of microgliosis, have increased H_2_O_2_-mediated chemoattraction towards α-syn aggregates (Ejlerskov et al., 2015), which can be enhanced by pre-injection of LPS in an oxidative-stress (rotenone) mouse model (Tien et al., 2013). Although a unified body of data is required to define a single model of MSA, the overproduction of cytotoxic by-products by microgliosis may contribute to α-syn misfolding/aggregation.

MSA may also share common pathological features with the tauopathy PSP, although astrocytes display a degenerative phenotype in PSP tissue rather than a reactive one (Togo and Dickson, 2002; Radford et al., 2015). Studies with microglial fractalkine receptor deficient hTau*Cx3cr1*^−/−^ mice have shown that enhanced microglial activation led to accelerated tau pathology and could be transferred to non-transgenic recipient mice by adoptive microglia; which was blocked by the interleukin1 receptor antagonist, Kineret (Bhaskar et al., 2010; Maphis et al., 2015). Recently, microglia have been shown to be directly linked to hTau propagation between non-synaptically connected neuronal populations *in vivo* via exosome release following phagocytosis of hTau (Asai et al., 2015). As most experiments to date have used models of PD to address α-syn diseases, future experiments will need to focus on specific MSA animal and cell culture models, such as by direct injection of purified GCI material (Radford et al., 2015), that may better mimic the disease’s specific cellular features (Halliday and Stevens, 2011), especially to further elucidate the role of both astrocytes and microglia in α-syn misfolding/aggregation and spreading.

## Neuroinflammation as a Therapeutic Target

Due to the possible cyclic nature of α-syn aggregation/release and gliosis in MSA, interventions that target neuroinflammation have the potential to slow the progression of disease and increase quality of life. Recent studies have approached α-synucleinopathies, including MSA, by use of immunotherapy (Valera and Masliah, 2013). For example, short immunogenic peptides mimicking the C-terminus of α-syn were administered to MBP-α-syn trangenic mice, followed by measurements of inducible anti-α-syn antibodies, cellular and tissue outcomes over time. Interestingly, as the peptides used did not carry the native epitope but instead a variation of it, this approach did not produce autoimmunity reactions via T-cells, leading to a beneficial prolonged response. Moreover, the induced antibodies were able to cross the blood-brain barrier where they could detect intracellular α-syn (monomer, oligomers and aggregates) after being internalized by microglia, oligodendrocytes and astrocytes. This reduced α-syn colocalization in oligodendrocytes and astrocytes but not in microglia, suggesting increased microglial uptake whilst preserving the oligodendrocyte population and decreasing demyelination, neuronal death and motor deficits (Mandler et al., 2015). Indeed, as in animal models of α-synucleinopathy the microglial response occurs prior to neuronal loss (Sanchez-Guajardo et al., 2015), strategies such as pre-immunization with α-syn peptides or approaches aimed at priming the CNS against α-syn immune insult may provide a glial memory and lessen subsequent neuroinflammatory responses to therapeutic benefit. Moreover, as activated microglia could participate actively in the spread of α-syn pathology, therapies that promote microgliosis to facilitate the clearance of extracellular pathological protein aggregates may have unwanted side effects. Furthermore, a recent study has shown that peripheral vector administration of the protease neurosin could degrade extracellular α-syn and thereby may reduce microglial and astrocyte activation (Spencer et al., 2015).

Some studies also aimed at microgliosis as a therapeutic target. For example, the inhibition of pro-inflammatory (iNOS or NAPH oxidase) enzymes from activated microglia is followed by decreased degeneration of neurons upon treatments with 7-nitroindazole and apocynin (Gao et al., 2003). Also, the treatment of the PLP-α-syn mouse model with minocycline decreased the density of activated microglia in the SNpc, reduced iNOS and TLR4-immunoreactivity and reduced dopaminergic degeneration (Stefanova et al., 2007).

As their differentiation capacity and immunomodulatory properties can address both neurodegeneration and neuroinflammation, mesenchymal stem-cells (MSCs) have been investigated as therapeutic options. In a study using the transgenic PLP mouse model of MSA (Stemberger et al., 2011), intravenous transplantation of MSCs promoted neuroprotection in the SNpc (as determined by TH+ neurons in the treated group) and down regulation of cytokines (IL-1α, IL-2, Il-10, TGF-β1 and TNF-α) in midbrain-brainstem lysates 4 weeks post-injection. Similarly, in the double-toxin-induced MSA-P mouse model, treatment with human MSC (hMSC) improved motor behavior (pole-descending test), increased neuronal survival (TH- and NeuN-positive markers), and decreased astro- and microgliosis (anti-Iba1 and anti-GFAP immunostaining, respectively) in the SNpc and striatum (Park et al., 2011). Lastly, a clinical trial using autologous MSC transplantation in patients with MSA-C improved symptoms severity [as observed on the baseline unified MSA rating scale (UMSARS)], and attenuated the declines of cerebral glucose metabolism and gray matter density (assessed by neuroimaging) along a 360-day follow-up (Lee et al., 2012). The immunosuppressive actions of MSC are believed to operate in a non-MHC-restricted manner, for example via secretion of soluble factors such as TGF-β1, PGE2 and indoleamine 2,3-dioxygenase (IDO; Krampera et al., 2006b). As glial cells are known to use IDO in the conversion of tryptophan to kynurenine, the finding that interferon-γ can upregulate the enzyme points to the kynurenine pathway as another therapeutic target in neuroinflammation related and non-related to MSCs (Krampera et al., 2006a). Recently, mice lacking anti-inflammatory interferon-β were shown to develop motor and cognitive deficits and α-syn pathology similar to a DLB/PD phenotype (Ejlerskov et al., 2015). This strengthens the link between dysfunctional inflammation and α-synucleinopathies and indicates dysfunction in inflammation can induce α-syn toxicity and* vice versa*.

Therapies targeting neuroinflammation in other α-syn disease models may warrant investigation in MSA. One example is the induction of heat shock proteins (HSPs) which are known to act via multiple pathways such as protein misfolding, neuroinflammation and mitochondrial oxidative phosphorylation. Recently, a HSP inducer, carbenoxolone, was shown to decrease astrogliosis, pro-inflammatory cytokines and oxidative stress in a rotenone model of PD (Thakur and Nehru, 2015). Additionally, HSPs are intimately involved in the degradation of α-syn by CMA (Wong et al., 2013; Vijayakumaran et al., 2015) and their induction may provide dual benefits by reducing neuroinflammation and toxic α-syn aggregates. The renin-angiotensin system may also be targeted to reduce the microglial inflammatory response via angiotensin II antagonists (Labandeira-Garcia et al., 2011). Microglial β-nicotinamide adenine dinucleotide phosphate oxidase 2 inhibitors may be useful by decreasing microglial O_2_- generation (Zhang et al., 2014). Other alternatives may be to combat oxidative stress, such as by the naturally occurring Acetyl-L-Carnitine (Singh et al., 2015). Counterbalancing neuroinflammation in glia and immune cells with naturally occurring lipids or hormonal modulation (Herrera et al., 2015; Skaper et al., 2015) may also be promising therapeutic strategies for MSA.

## Conflict of Interest Statement

The authors declare that the research was conducted in the absence of any commercial or financial relationships that could be construed as a potential conflict of interest.

## Abbreviations

α-syn: α-synuclein
CMA: Chaperone-mediated autophagy
CNP: Cyclic nucleotide phosphodiesterase
CNS: Central nervous system
CSFs: Colony-stimulating factors
DLB: Dementia with Lewy bodies
ER: Endoplasmic reticulum
GCI: Glial cytoplasmic inclusion
GFAP: Glial fibrillary acidic protein
HSP: Heat shock protein
IDO: Indoleamine 2,3-dioxygenase
IL: Interleukin
iNOS: Inducible nitric oxide synthase
LPS: Lipopolysaccharide
MBP: Myelin basic protein
MSA: Multiple system atrophy
MSC: Mesenchymal stem-cells
NADPH: Nicotinamide adenine dinucleotide phosphate hydroxylase
NF-κB: Nuclear factor kappa B
NO: Nitric oxide
PD: Parkinson’s disease
PET: Positron Emission Tomography
PGE2: Prostaglandin E2
PLP: Proteolipid protein
PSP: Progressive Supranuclear Palsy
ROS: Reactive oxygen species
SNARE: Soluble NSF Attachment Protein receptor
SNpc: Substantia nigra pars compacta
TGF: Transforming growth factor
TH: Tyrosine hydroxylase
TLR: Toll-like receptors
TNF: Tumour necrosis factor
YFP: Yellow fluorescent protein.
